# Long Non-coding RNAs LOC100126784 and POM121L9P Derived From Bone Marrow Mesenchymal Stem Cells Enhance Osteogenic Differentiation via the miR-503-5p/SORBS1 Axis

**DOI:** 10.3389/fcell.2021.723759

**Published:** 2021-10-22

**Authors:** Yiyang Xu, Ruobing Xin, Hong Sun, Dianbo Long, Zhiwen Li, Hongyi Liao, Ting Xue, Ziji Zhang, Yan Kang, Guping Mao

**Affiliations:** ^1^Department of Joint Surgery, The First Affiliated Hospital, Sun Yat-sen University, Guangzhou, China; ^2^Guangdong Provincial Key Laboratory of Orthopedics and Traumatology, Guangzhou, China; ^3^Department of Orthopedics, Shengli Clinical Medical College, Fujian Provincial Hospital, Fujian Medical University, Fuzhou, China; ^4^Department of Orthopaedics, Affiliated Hospital of Guizhou Medical University Guiyang, Guizhou, China; ^5^Fujian Provincial Hospital South Branch, Center of Health Management, Shengli Clinical Medical College of Fujian Medical University, Fuzhou, China

**Keywords:** LOC100126784, POM121L9P, miR-503-5p, sorbin and SH3 domain containing-1 gene, bone marrow mesenchymal stem cell, osteogenesis

## Abstract

Long non-coding RNAs (lncRNAs) play pivotal roles in mesenchymal stem cell differentiation. However, the mechanisms by which non-coding RNA (ncRNA) networks regulate osteogenic differentiation remain unclear. Therefore, our aim was to identify RNA-associated gene and transcript expression profiles during osteogenesis in bone marrow mesenchymal stem cells (BMSCs). Using transcriptome sequencing for differentially expressed ncRNAs and mRNAs between days 0 and 21 of osteogenic differentiation of BMSCs, we found that the microRNA (miRNA) miR-503-5p was significantly downregulated. However, the putative miR-503-5p target, sorbin and SH3 domain containing 1 (SORBS1), was significantly upregulated in osteogenesis. Moreover, through lncRNA-miRNA-mRNA interaction analyses and loss- and gain-of-function experiments, we discovered that the lncRNAs LOC100126784 and POM121L9P were abundant in the cytoplasm and enhanced BMSC osteogenesis by promoting SORBS1 expression. In contrast, miR-503-5p reversed this effect. Ago2 RNA-binding protein immunoprecipitation and dual-luciferase reporter assays further validated the direct binding of miR-503-5p to LOC100126784 and POM121L9P. Furthermore, SORBS1 knockdown suppressed early osteogenic differentiation in BMSCs, and co-transfection with SORBS1 small interfering RNAs counteracted the BMSCs’ osteogenic capacity promoted by LOC100126784- and POM121L9P-overexpressing lentivirus plasmids. Thus, the present study demonstrated that the lncRNAs LOC100126784 and POM121L9P facilitate the osteogenic differentiation of BMSCs via the miR-503-5p/SORBS1 axis, providing potential therapeutic targets for treating osteoporosis and bone defects.

## Introduction

Bone marrow mesenchymal stem cells (BMSCs) are ubiquitous, totipotent, and self-renewing. They have multidirectional differentiation potential and can undergo osteogenesis, chondrogenesis, and adipogenesis (Pittenger et al., 1999). BMSCs have significantly higher osteogenic capacity than adipose-derived stem cells. They exhibit low immunogenicity and a capacity for direct homing (Chen et al., 2016; Mohamed-Ahmed et al., 2018). Hence, BMSCs are promising for bone tissue engineering and have been preliminarily studied in animal models (Arthur et al., 2009; Elsafadi et al., 2016; Ho-Shui-Ling et al., 2018; Arthur and Gronthos, 2020). However, BMSC grafting may be ineffective over time because of the associated reduction in proliferation and osteoblastic differentiation ability (Derubeis and Cancedda, 2004; Ho-Shui-Ling et al., 2018). Therefore, identifying the molecular mechanisms by which BMSCs are selected for osteogenic differentiation may enable the elucidation of ways to maintain their osteogenic ability.

Long non-coding RNAs (lncRNAs) are long transcripts of up to 200 nucleotides (nt) (Mercer et al., 2009) that lack any apparent protein-coding function (Quinn and Chang, 2016). The major functions of lncRNAs are transcription and post-transcriptional regulation (Dykes and Emanueli, 2017). MicroRNAs (miRNAs) are short non-coding RNAs less than 22 nt in length. They negatively regulate mRNA expression by targeting short 7-nt seeds sequenced by the miRNA response element (MRE) (Fabian and Sonenberg, 2012). It was hypothesized that competing endogenous RNAs (ceRNAs) enable lncRNAs and miRNAs to form extensive regulatory networks in cells (Salmena et al., 2011). Previous studies have indicated that lncRNAs play pivotal roles in osteogenesis (Schmitz et al., 2016; Uszczynska-Ratajczak et al., 2018; Yang et al., 2018; Silva et al., 2019; Lanzillotti et al., 2021); they regulate osteoblastic differentiation through canonical signaling pathways and key transcription factors. However, the molecular functions and mechanisms of lncRNA-miRNA-mRNA networks in the early stages of osteogenesis in BMSCs have not been fully clarified.

The sorbin homology (SoHo) family of adapter and scaffold proteins consists of three proteins: c-Cbl associated protein (CAP), also known as Sorbin and SH3 domain containing 1 (SORBS1), ArgBP2 (SORBS2), and Vinexin (SORBS3) (Ichikawa et al., 2017). Previous reports have suggested that SORBS1 encodes an insulin-signaling molecule mainly expressed in adipose and skeletal muscles, affects actin cytoskeleton organization, and modulates the location of focal adhesion complex, acting as a unique stiffness-mediated sensor for osteogenic differentiation (Hong et al., 2005; Engler et al., 2006; Wada et al., 2011; Kuroda et al., 2018). CAP protein encoded by the SORBS1 gene, combined with vinculin in the integrin complex, mediates homeostatic adaptation to external forces (Carisey and Ballestrem, 2011; Bharadwaj et al., 2013; Chen and Jacobs, 2013). Reports from different groups have confirmed that SORBS1 knockdown inhibits the osteogenic differentiation process of stem cells (Holle et al., 2013, 2016; Kuroda et al., 2018). The present study used RNA sequences to profile gene and transcript expression associated with these RNAs during osteogenesis in BMSCs. SORBS1 showed a significant difference during this induction of osteogenic differentiation. However, there is no doubt that this is a complex and unclear regulatory network involving calcium, mitogen-activated protein kinase/extracellular signal-regulated kinase, Wnt, and Yes-associated protein/transcriptional coactivator with PDZ-binding motif pathways (Bharadwaj et al., 2013; Chen and Jacobs, 2013; Holle et al., 2016; Vdovenko et al., 2020). Therefore, we further investigated whether LOC100126784 and POM121L9P/miR-503-5p regulate SORBS1 and modulate early osteogenic differentiation in BMSCs.

## Materials and Methods

### Isolation, Culture, and Multidirectional Differentiation of Bone Marrow Mesenchymal Stem Cells

Bone marrow samples were obtained from the First Affiliated Hospital of Sun Yat-Sen University. Bone marrow samples were collected from six healthy adult donors (three males with an average age of 34 years and three females with an average age of 32 years). All procedures were approved by the Ethical Committee of the First Affiliated Hospital of Sun Yat-Sen University (IRB: 2014C-028) and complied with the Declaration of Helsinki (WMA, 2000). All volunteers provided written informed consent. BMSCs were isolated as previously described (Mao et al., 2018; Sun et al., 2018). The cells were cultured in alpha-modified Eagle’s medium (α-MEM; Gibco, Grand Island, NY, United States) supplemented with 10% (v/v) fetal bovine serum (FBS; Gibco, Grand Island, NY, United States), 100 U/mL penicillin, and 100 μg/mL streptomycin (Gibco, Grand Island, NY, United States) and incubated at 37°C in a 5% CO_2_ atmosphere. The cell culture medium was changed every 3 days, and the cells were detached with 0.05% trypsin-ethylenediaminetetraacetic acid (EDTA) at 80% confluency. For osteogenic differentiation, BMSCs at the third passage were seeded in six-well plates (NEST Biotechnology Co., Ltd., Wuxi, China) at a density of 2 × 10^4^/cm^2^. Each well contained 2 mL complete medium (Cyagen Biosciences, Guangzhou, China).

The chondroblast and adipoblast differentiation methods were consistent with those previously reported (Mao et al., 2018). The chondrogenic induction medium comprised a complete medium supplemented with 1:100 transforming growth factor (TGF-β3; Cyagen Biosciences, Guangzhou, China).

### RNA Isolation, Microarray Hybridization, and Bioinformatics Analysis

Total RNA was extracted from the BMSCs with TRIzol reagent (Invitrogen, Carlsbad, CA, United States). cDNA libraries were constructed for each pooled RNA sample using a NEBNext^®^ Ultra^TM^ Directional RNA Library Prep Kit for Illumina (New England Biolabs, Ipswich, MA, United States). A NEBNext^®^ Small RNA Library Prep Set for Illumina (New England Biolabs, Ipswich, MA, United States) was used to generate the miRNA sequencing library. The libraries were purified by gel electrophoresis, their qualities were controlled using TapeStation 2200 (Agilent Technologies, Santa Clara, CA, United States), and they were sequenced with HiSeq Xten (Illumina, San Diego, CA, United States). Clean reads were aligned to Human Genome v. GRCh38 (NCBI, Bethesda, MD, United States) using HISAT2. A differential transcript analysis of the RNA-seq experiments via HTSeq was used to count the mRNA and lncRNA reads. The miRNA-Seq reads were filtered and mapped using BWA aln to Human miRNA Database (miRbase) v. 22.0 and Human Genome v. GRCh38 to achieve miRNA expression. Finally, we utilized the Miranda and RNAhybrid databases to predict the miRNA targets on lncRNA and mRNA.

### RNA Extraction, Reverse Transcription, and RT-qPCR

Total cellular RNA was extracted using the miRNA Mini Kit (Qiagen, Hilden, Germany) according to the manufacturer’s protocol. Nuclear RNA and cytoplasmic RNA were extracted following the instructional manuals of the nuclear and cytoplasmic extraction reagents kit (Beyotime, Beijing, China). cDNA was synthesized from miRNA and mRNA using the Mir-X^TM^ miRNA First-Strand Synthesis Kit (Takara Bio Inc., Shiga, Japan) and a PrimeScript^TM^ RT Master Mix (Takara Bio Inc., Shiga, Japan), respectively. PCR reaction was conducted in 20 μl total volume containing a final concentration of 0.5 mM of each primer, 6.4 μl ddH_2_O, 10 μl of 2x SYBR Premix Ex Taq^TM^ II (Takara Bio Inc., Shiga, Japan) and 2 μl of cDNA sample (1:20 diluted) corresponding to 1 μg of total RNA. RT-qPCR reaction was performed on an ABI ViiA^TM^ 7 Real-Time PCR System (Applied Biosystems, Foster City, CA, United States). The amplification products were detected by agarose gel electrophoresis and sequencing. Transcript levels of lncRNA and mRNA were normalized to that of the GAPDH housekeeping gene, and those of miRNA were normalized to that of the small U6 RNA. The RT-qPCR primer sequences are listed in Supplementary Table 1. Expression levels were calculated using the 2^–ΔΔCt^ method (Livak and Schmittgen, 2001). Each experiment was performed in triplicate.

### Western Blotting

Cellular proteins were extracted, and western blotting was performed as previously reported (Mao et al., 2017). Briefly, total proteins were isolated from BMSCs with radioimmunoprecipitation assay (RIPA) buffer (Beyotime Biotechnology, Beijing, China) containing protease inhibitors (Abcam, Cambridge, United Kingdom). Membranes were incubated at 4°C overnight with primary antibodies against RUNX2 (1:2,000; Cell Signaling Technology, Danvers, MA, United States), OCN, OPN, and SORBS1 (1:1,000; Proteintech, Wuhan, China). GAPDH (1:3,000; Cell Signaling Technology, Danvers, MA, United States) was used as the internal control. Appropriate secondary antibodies (1:3,000; Cell Signaling Technology, Danvers, MA, United States) were incubated with the blots at 20–25°C for 1 h. After rinsing with Tris-buffered saline (TBS) containing 0.05% (w/v) Tween-20 (TBST), the signals were detected using an enhanced chemiluminescence (ECL) kit (Millipore, Darmstadt, Germany) and a chemiluminescence system (Bio-Rad Laboratories, Hercules, CA, United States) and analyzed with Image Lab v6.0 software.^1^

### Animal Model and Immunohistochemical Analysis

All procedures were approved by the First Affiliated Hospital of Sun Yat-sen University ([2013]A-110) Animal Research Committee. Femur samples were used to examine target molecule expression in either young (8-week) or aged (18-month) male C57BL/6 mice (Piemontese et al., 2017). IHC analysis was performed according to a previously reported method (Sun et al., 2018). Briefly, the femur samples were immersed in 4% (v/v) paraformaldehyde (Sigma-Aldrich Corp., St. Louis, MO, United States), decalcified in 10% EDTA for 2 weeks, embedded in paraffin, and cut into 5-μm-thick sections using Leica 2235 (Leica Biosystems, Nussloch, Germany). Then, sections were incubated with sodium citrate buffer at 99°C for 20 min for antigen retrieval and rabbit anti-SORBS1 (1:200; Proteintech, Wuhan, China) afterward for IHC analysis.

### Multipotential Differentiation and Flow Cytometry Analyses

Differentiation was induced in BMSCs, and they were cultured as described previously (Li et al., 2020). Lipid vesicles in adipogenically induced BMSCs were stained with Oil Red O. The degree of osteogenic differentiation was determined using Alizarin Red staining. The cartilaginous phenotype was confirmed using Alcian Blue staining (Cyagen Biosciences). Flow cytometry was performed to identify the CD29, CD44, and CD90 surface antigens of BMSCs and the hematopoietic CD34 cell lines. Monoclonal antibodies (1:100; BioLegend Inc., San Diego, CA, United States) against the antigens mentioned above were used for detection. Mouse/rabbit IgG monoclonal antibody (BioLegend Inc.) was used as the negative control.

### Luciferase Reporter Assay

BMSCs were seeded in 24-well plates and cultured to 60–80% confluency. For SORBS1/miR-503-5p, either wild type (WT) or mutant SORBS1 3′ UTR fragments (512 bp) were inserted into a pSI-Check2 luciferase vector (Hanbio, Shanghai, China). The cells were transfected with 800 ng SORBS1 3′UTR-wt or SORBS1 3′UTR-mut plasmids, 20 nmol miR-503-5p mimics, and a control. For LOC100126784/miR-503-5p and POM121L9P/miR-503-5p, either WT or mutant LOC100126784 fragments (500 bp) and WT or mutant POM121L9P fragments (495 bp), respectively, were inserted into a pSI-Check2 luciferase vector (Hanbio). The cells were transfected with 1 μg of LOC100126784-wt, LOC100126784-mut, POM121L9P-wt, or POM121L9P-mut plasmids, 20 nmol miR-503-5p mimics, and a control. After 48 h of incubation, a Firefly and Renilla-Glo Luciferase Reporter Assay Kit (Meilunbio, Dalian, China) was used to measure relative luciferase activity.

### RNA Fluorescent *in situ* Hybridization

The FISH assay was performed in BMSCs. Slides containing BMSCs were fixed with 4% paraformaldehyde for 20 min. After prehybridization using 1 × PBS and 0.5% Triton X-100 for 1 h, hybridization was performed overnight with specific probes at 42°C. The slides were washed thrice using 2, 1, and 0.5 × SSC buffer, respectively. The images were acquired via confocal microscopy (Zeiss LSM710, Oberkochen, Germany). Nuclei were counterstained with 2 mg/mL 4,6-diamidino-2-phenylindole (DAPI) for 8 min at 20–25°C. The biotin-labeled probes for lncRNAs were synthesized from Servicebio (Wuhan, China). The probe sequences are listed as below: POM121L9P probe:5′-TCGCAGAGGGCATCGGTGAGCATTGGA-3′; LOC 100126784 probe: 5′-GGTTCGACGCCCCCTAGCCTGGCTG TAAGAT-3′.

### Transfection

Human full-length LOC100126784 cDNA (3753 bp, NR_015384.2) and POM121L9P cDNA (5224 bp, NR_003714.1) were produced using a standard PCR protocol, and their overexpression vectors were constructed by OBiO Technology (Shanghai, China) as described previously (Mao et al., 2018). BMSCs were transfected with miR-503-5p mimics (50 nM; RiboBio, Guangzhou, China), SORBS1 small interfering RNAs (100 nM; RiboBio), pSLenti-LOC100126784 plasmids (500 ng; OBiO Technology, Shanghai, China), and pSLenti-POM121L9P plasmids (500 ng; OBiO Technology) using Lipofectamine^TM^ 3000 (Invitrogen). Cells were collected after 48 h for RT-qPCR analysis and after 72 h for western blotting and immunofluorescence analysis. The sequences are shown as follows: hsa-miR-503-5p mimics: 5′-UAGCAGCGGGAACAG UUCUGCAG-3′, 5′-CUGCAGAACUGUUCCCGCUGCUA-3′; hsa-miR-503-5p inhibitor: 5′-CUGCAGAACUGUUCCCGCU GUA-3′; siSORBS1#1: CCGGAAATTTCTTCAGAGA; siSOR BS1#2: CCAGTCCTCTACTAAATGA; siSORBS1#3: 5′-GAGCC AAATTTGACTTTAA-3′; siRNA-POM121L9P#1: 5′-CAACA CTCACTGACATCGA-3′; siRNA-POM121L9P#2: 5′-CAAGG GCAGCAGTGCACAT-3′; siRNA-POM121L9P#3: 5′-GGCCG GACACGTGGTAGAT-3′; siRNA-LOC100126784#1: 5′-GGTC TCGAAGAGCTCTTAA-3′; siRNA-LOC100126784#2: 5′-GCT TACATGCATGCTTATA-3′; siRNA-LOC100126784#3: 5′-GCATCCTAGTCTCAGACTT-3′. Non-specific miRNA (miR- Control, RiboBio) and siRNA-Control (RiboBio) were used as negative controls.

### RNA-Binding Protein Immunoprecipitation Assay

An Ago2-RIP assay was conducted using the Magna RIPTM RNA-Binding Protein Immunoprecipitation Kit (EMD Millipore, Bedford, MA, United States). Approximately 1 × 10^7^ lysed BMSCs were serially treated with a combination of RIP lysis buffer, protease inhibitor cocktail, RNase inhibitors, and RNase-free DNase. Resuspended RNA complexes were used for immunoprecipitation at 4°C overnight in the presence of anti-Ago2 antibody (1:50; Abcam, Cambridge, United Kingdom) or mouse IgG-coated magnetic beads included in the kit. Absorbed RNAs were treated with proteinase K buffer and purified with TRIzol reagent for cDNA synthesis. RT-qPCR was used to detect specific RNA expression with the sequence-specific primers mentioned above.

### Immunofluorescence

BMSCs were grown in 20 mm glass-bottom cell culture dishes (NEST, Wuxi, China). The cells were fixed with 4% (v/v) paraformaldehyde for 20 min, washed thrice with phosphate-buffered saline (PBS) containing 0.05% (w/v) Tween-20 (PBS-T), permeabilized with 0.3% (w/v) Triton X-100 for 5 min, and blocked with 1% (v/v) bovine serum albumin for 30 min. The cells were incubated at 4°C overnight with antibodies against OCN, OPN, and RUNX2 (1:100; Proteintech, Wuhan, China). The cells were washed thrice with PBS and incubated with goat anti-rabbit IgG conjugated with fluorescent cy5 dye (1:100; Abcam) in PBS. Then, DAPI (Life Technologies, Carlsbad, CA, United States) was used for nuclear staining. Immunofluorescence images were obtained with a Zeiss LSM710 confocal laser scanning microscope (Carl Zeiss AG, Oberkochen, Germany) and analyzed using ImageJ v.1.5a (NIH, Bethesda, MD, United States).

### Statistical Analysis

All experiments were performed using at least three biological replicates. Comparisons between two groups were performed using unpaired two-tailed Student’s *t*-test, Welch’s *t*-test (unequal variances), or Mann–Whitney *U*-test (non-normal distribution). Data were processed using SPSS v. 20.0 (IBM Corp., Armonk, NY, United States). Statistical significance was set at *p* < 0.05.

## Results

### LncRNA, miRNA, and mRNA Expression Profiles During Osteogenic Bone Marrow Mesenchymal Stem Cell Differentiation

**FIGURE 1:**
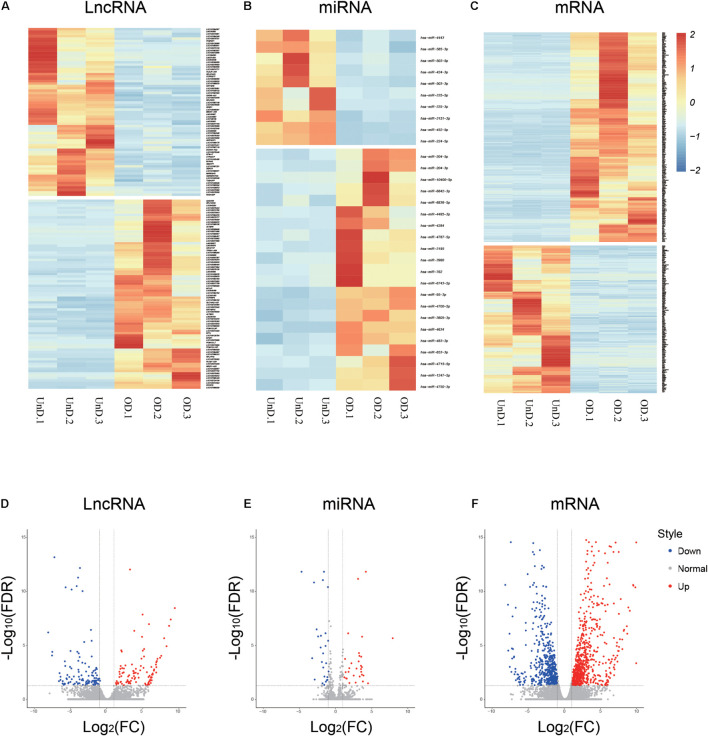
Differential expression profiles of long non-coding RNAs (lncRNAs), microRNA (miRNAs), and mRNAs during osteogenic differentiation. Heat maps **(A–C)** and volcano plots **(D–F)** showing differential ncRNA and mRNA expression levels between osteogenic-differentiated bone marrow mesenchymal stem cells (BMSCs) and undifferentiated BMSCs.

The RNA-seq analysis provided an overview of the lncRNAs, miRNAs, and mRNAs differentially expressed in osteogenic-differentiated BMSCs (day 21) compared with undifferentiated BMSCs (day 0). A total of 80 upregulated and 71 downregulated lncRNAs were identified during the osteogenic-induced BMSCs (log_2_ fold change > 2.0, *p* < 0.05; Figure 1A). There were 21 upregulated and 10 downregulated miRNAs in the osteogenically differentiated BMSCs (Figure 1B). In addition, analysis of mRNA levels revealed that 463 mRNAs were upregulated and 325 mRNAs were downregulated in BMSCs during this process (Figure 1C). Corresponding volcanic plots for differential expression of the lncRNAs (Figure 1D), miRNAs (Figure 1E), and mRNAs (Figure 1F) are shown. All transcript expression data are presented in Supplementary Table 2.

### LncRNA-miRNA-mRNA Co-expression Network Construction and Bioinformatic Analysis

**FIGURE 2:**
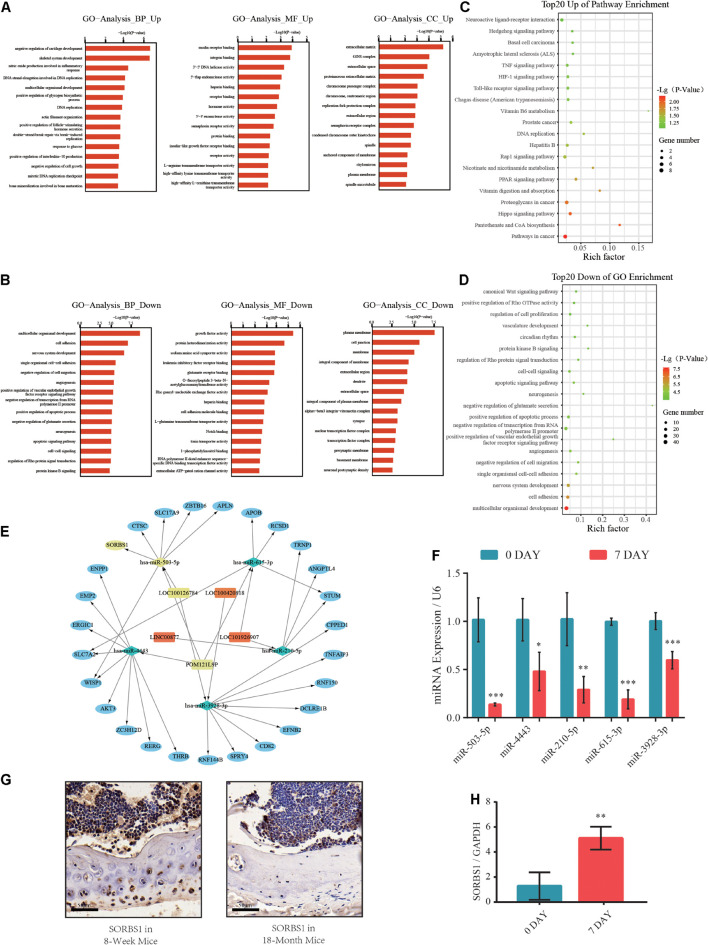
Gene ontology (GO) and Kyoto Encyclopedia of Genes and Genomes (KEGG) pathway analyses of differentially expressed genes (DEGs) in competing endogenous RNA (ceRNA) network. **(A,B)** Significantly upregulated and downregulated GO annotations with top 20 scores. **(C,D)** Significantly upregulated and downregulated KEGG pathway annotations with top 20 scores. **(E)** Construction of predicted miRNA-mRNA network core in osteogenic differentiation. **(F)** Relative expression levels of miR-503-5p, miR-4443, miR-210-5p, miR-615-3p, and miR-3928-3p used to distinguish between undifferentiated (day 0) and osteogenically differentiated (day 7) groups. **(G)** SORBS1 levels in femoral cortices and bone marrow of young mice (8 weeks) and aged mice (18 months) as determined using immunohistochemistry (IHC). Scale bar, 50 μm. **(H)** Relative expression levels of SORBS1 in undifferentiated (day 0) and osteogenically differentiated (day 7) groups, as measured by RT-qPCR. GAPDH served as an endogenous control for mRNAs, and U6 acted as an endogenous control for miRNAs. Quantitative data are presented as mean ± standard deviation (SD) of three independent experiments; *p < 0.05, **p < 0.01, ***p < 0.001.

Based on the Miranda and RNAhybrid databases and differential expression of lncRNAs, miRNAs, and mRNAs, we obtained a differentially expressed ceRNA network comprising 125 lncRNAs, 41 miRNAs, and 431 mRNAs (Supplementary Table 3). Gene ontology (GO) analysis categorized the differentially expressed transcripts as biological processes (BP), cellular components (CC), and molecular functions (MF). A GO analysis of the upregulated genes revealed that negative regulation of cartilage development and skeletal system development were enhanced (Figure 2A). However, the downregulated GO terms showed attenuation of multicellular organismal development, growth factor activity, and plasma membrane (Figure 2B). Kyoto Encyclopedia of Genes and Genomes (KEGG) pathway analysis revealed significantly upregulated and downregulated terms such as Hippo, Hedgehog, organismal development, and cell adhesion signaling pathways (Figures 2C,D). The ncRNA-mRNA networks were constructed by the markedly expressed mRNAs in GO analysis and their putative upstream ncRNAs (Figure 2E). Moreover, we found that the expression of miR-503-5p was significantly lower in osteogenic-differentiated BMSCs than in undifferentiated BMSCs (Figure 2F). Furthermore, IHC revealed that SORBS1 protein expression was significantly higher in the femoral cortices of young developing mice (8 weeks) than in those of aged mice (18 months) (Figure 2G). RT-qPCR showed that SORBS1 was upregulated in osteoblast-induced BMSCs (Figure 2H). Thus, SORBS1 upregulation and miR-503-5p downregulation may have a constituent ceRNA-based relationship that needs to be further explored.

### MiR-503-5p Regulated SORBS1 Expression in Bone Marrow Mesenchymal Stem Cells During Osteogenesis

**FIGURE 3:**
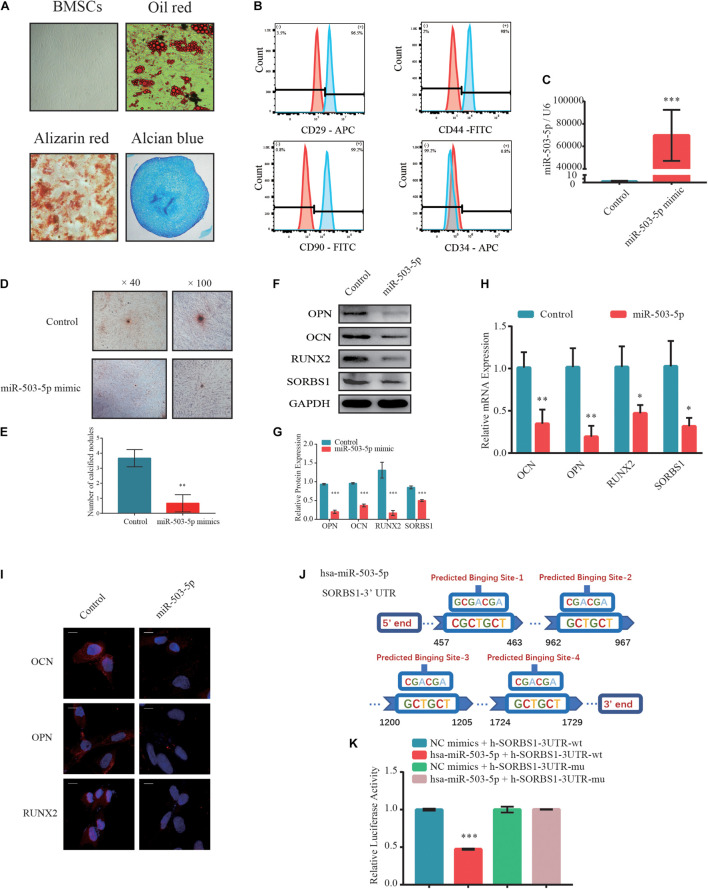
MiR-503-5p/SORBS1 selected to verify the feasibility of the predicted ceRNA network. **(A)** Adipogenesis, osteogenesis, and chondrogenesis potentials demonstrated by Oil Red O, Alizarin Red, and Alcian Blue staining, respectively. **(B)** BMSCs identified using flow cytometry. Positive mesenchymal markers included CD29, CD44, and CD90, while the negative mesenchymal marker was CD34. Light blue histograms denote isotype controls. Light red peaks indicate tested markers. **(C)** MiR-503-5p transfection efficiency as assessed by RT-qPCR. **(D)** Effects of miR-503-5p on mineralized nodule formation, as demonstrated by Alizarin Red staining and **(E)** quantitative analysis of mineralized nodules. Expression levels of osteogenesis markers OCN, OPN, and RUNX2 and targeted gene SORBS1 in transfected BMSCs detected using western blotting **(F)**, quantified protein expression by densitometric analysis **(G)**, RT-qPCR **(H)**, and immunofluorescence **(I)**. **(J)** Potential binding sites between miR-503-5p and SORBS1-3’ UTR. **(K)** miR-503-5p binds SORBS1-3’ UTR, as confirmed by luciferase activity assay. GAPDH served as an endogenous control for mRNAs, and U6 acted as an endogenous control for miRNAs. Quantitative data of three independent samples are presented. ^∗^*p* < 0.05, ^∗∗^*p* < 0.01, ^∗∗∗^*p* < 0.001.

Next, the BMSCs at passage 3 (P3) were identified by their multidirectional differentiation potential and flow cytometry. Adipogenic potential was assessed by observing small cytoplasmic lipid droplets stained with Oil Red O. Osteogenic potential was evaluated by staining calcium mineral deposits with Alizarin Red. Chondrogenic potential was verified by sectioning beads and staining sulfated glycosaminoglycans with Alcian Blue (Figure 3A). Flow cytometry revealed that the positive markers CD29, CD44, and CD90 and the negative CD34 phenotype indicated successful BMSC isolation (Figure 3B).

To determine whether miR-503-5p regulates SORBS1 expression in BMSCs, we transfected these cells with miR-503-5p or miR-control and validated transfection efficiency by RT-qPCR after 48 h (Figure 3C). There were relatively fewer and smaller calcium nodules stained with Alizarin Red in response to miR-503-5p overexpression rather than control group on day 7 after osteogenic induction (Figure 3D). Quantitative analysis showed significant differences in the number of calcium nodules (Figure 3E). Similar protein expression patterns were identified by western blotting (Figures 3F,G) and immunofluorescence analysis (Figure 3I). RT-qPCR analysis showed that miR-503-5p downregulated the expression of osteogenic markers OCN, OPN, and RUNX2, as well as the putative target, SORBS1 (Figure 3H). Furthermore, miR-503-5p knockdown by transfection with an miRNA-inhibitor was confirmed by RT-qPCR (Supplementary Figure 1A). The decreased miR-503-5p expression promoted the upregulation of osteogenic markers and SORBS1 at the mRNA and protein levels (Supplementary Figures 1D,G,J).

To further investigate the interaction between miR-503-5p and SORBS1, we constructed wild-type (WT) and mutant (MUT) SORBS1-3′ UTR dual-luciferase reporter plasmids (Figure 3J). The luciferase assay revealed that miR-503-5p transfection significantly suppressed luciferase activity in BMSCs transfected with SORBS1 WT-3′ UTR but not in those transfected with SORBS1 MUT-3′ UTR (Figure 3K). These findings indicate that miR-503-5p attenuates osteogenic differentiation and the expression of SORBS1 mRNA and protein.

### LOC100126784 and POM121L9P Enhanced Bone Marrow Mesenchymal Stem Cell Osteogenesis by Promoting SORBS1 Expression

**FIGURE 4:**
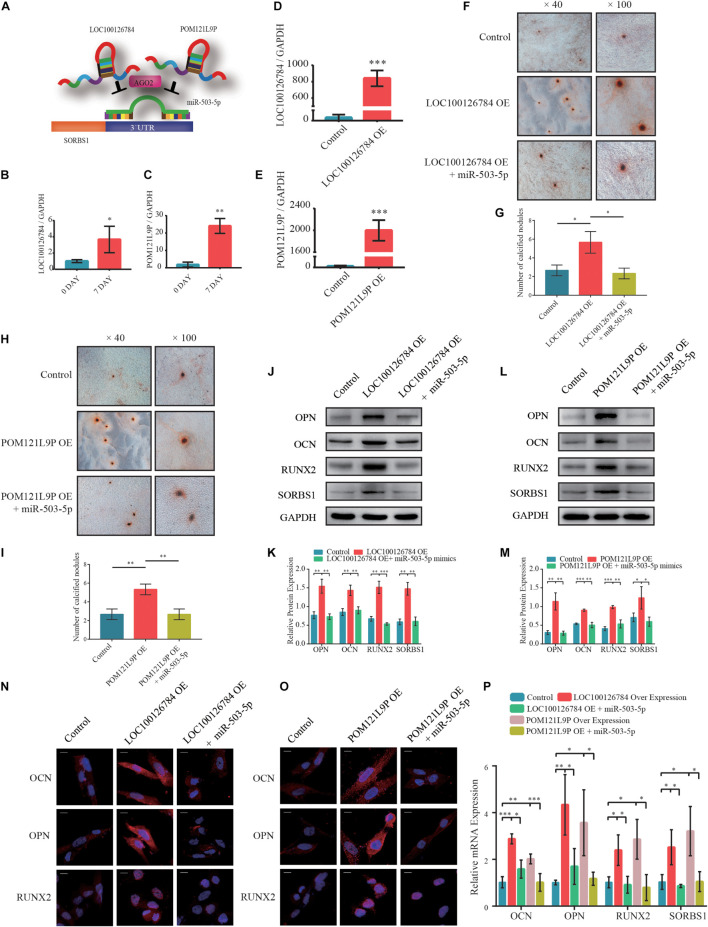
LOC100126784 and POM121L9P promotes osteogenic differentiation in BMSCs by inhibiting miR-503-5p. **(A)** Schematic illustration of LOC100126784 and POM121L9P sponging of SORBS1-targeting miR-503-5p. **(B,C)** Verification of relative RNA expression levels of LOC100126784 and POM121L9P during early osteoblast differentiation of BMSCs. **(D,E)** BMSCs were transfected with lentiviral or empty vector and miRNA- or control-mimics. LOC100126784 and POM121L9P expression levels were measured using RT-qPCR. **(F,H)** On day 7 of osteogenic induction in BMSCs, slides were stained with Alizarin Red. Calcium node size and number were evaluated at × 40 and × 100 magnification. **(G,I)** Quantitative analysis of the number of calcium nodules in **(F,H)**. Expression levels of osteogenesis markers OCN, OPN, and RUNX2 and the targeted gene SORBS1 were detected via western blotting **(J,L)**, quantified protein expression by densitometric analysis **(K,M)**, immunofluorescence (IF) **(N,O)**, and RT-qPCR **(P)**. GAPDH served as an endogenous control. Quantitative data are presented as mean ± SD of three independent experiments; ^∗^*p* < 0.05, ^∗∗^*p* < 0.01, ^∗∗∗^*p* < 0.001.

To explore the upstream target of miR-503-5p, RNAhybrid and Miranda databases were used to predict putative miR-503-5p binding sites; the results indicated that LOC100126784 and POM121L9P sponge miR-503-5p (Figure 4A). RT-qPCR analysis verified the early expression of LOC100126784 and POM121L9P during osteogenic BMSC differentiation (Figures 4B,C).

We used RT-qPCR to determine transfection efficiency in LOC100126784- and POM121L9P-overexpressing (OE) lentivirus plasmids (Figures 4D,E). We co-infected BMSCs with OE-LOC100126784, OE-POM121L9P, control plasmids, miR-503-5p, and miR-control to identify any inhibitory relationships among them. We evaluated the extent of Alizarin Red staining on day 7 of osteogenic induction. LOC100126784 and POM121L9P overexpression produced relatively more and larger calcium nodules in BMSCs. Interestingly, these trends were attenuated by co-transfection with miR-503-5p mimics (Figures 4F,H). The number of calcium nodules was determined by quantitative analysis (Figures 4G,I). The protein (Figures 4J–M) levels of OCN, OPN, RUNX2, and SORBS1 were significantly lower in BMSCs co-transfected with OE-LOC100126784 or OE-POM121L9P and miR-503-5p mimics than in those transfected with OE-LOC100126784 or OE-POM121L9P alone. Immunofluorescence analysis confirmed that OCN, OPN, and RUNX2 were upregulated in BMSCs overexpressing either LOC100126784 or OE-POM121L9P. However, these osteogenic biomarkers returned to control levels after miR-503-5p co-transfection (Figures 4N,O). Similar changes in mRNA levels are shown in Figure 4P.

To select the most efficient small interfering RNAs for LOC100126784 and POM121L9P, RT-qPCR was used to detect LOC100126784 and POM121L9P expression levels after transfection (Supplementary Figures 1B,C). In addition, we examined the effects of LOC100126784 and POM121L9P knockdown on osteogenic differentiation. mRNA (Supplementary Figures 1E,F) and protein (Supplementary Figures 1H,I,K,L) levels of OCN, OPN, RUNX2, and SORBS1 were decreased after transfection with LOC100126784 and POM121L9P siRNAs but returned to control levels upon co-transfection with the miR-503-5p-inhibitor. Taken together, both LOC100126784 and POM121L9P promoted osteogenic differentiation in BMSCs, whereas miR-503-5p inhibited it.

### LOC100126784 and POM121L9P Independently Sponge miR-503-5p

**FIGURE 5:**
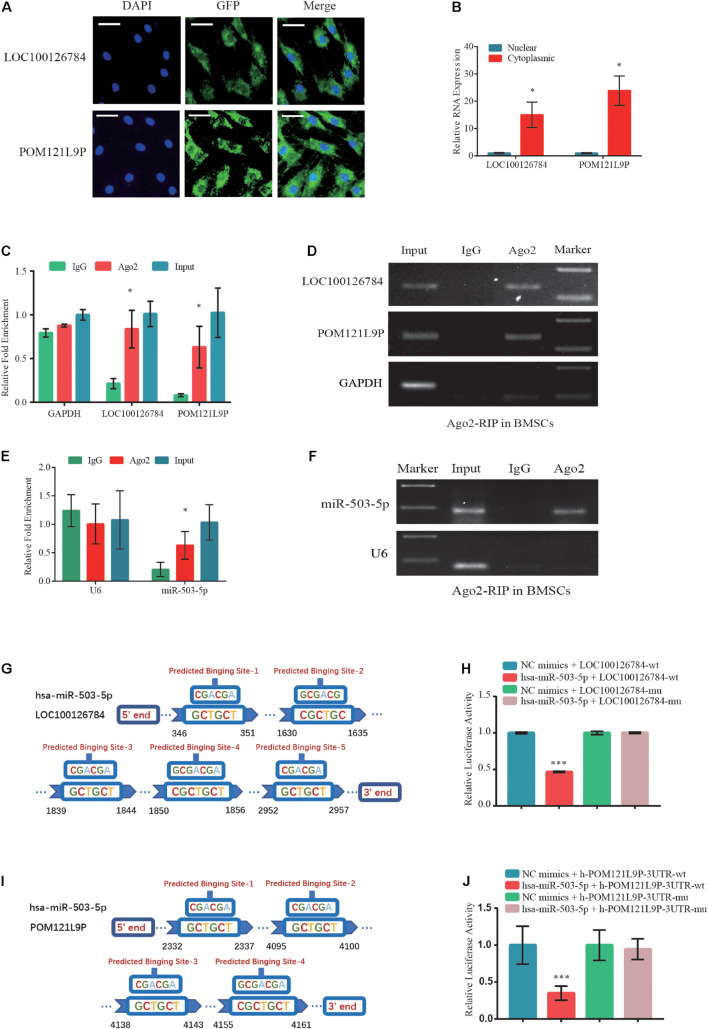
Direct LOC100126784 and POM121L9P binding to miR-503-5p in the cytoplasm. **(A)** RNA fluorescence *in situ* hybridization (FISH) images showing the localization of LOC100126784 and POM121L9P in BMSCs. LncRNAs probes were labeled with Alexa fluor 488, and nuclei were stained with DAPI. Scale bar, 50 μm. **(B)** RNA-binding protein immunoprecipitation (RIP) assay of Ago2 vs. IgG enrichment on LOC100126784, POM121L9P **(C,D)** and miR-503-5p **(E,F)** in BMSCs and detection by RT-qPCR and gel electrophoresis. Predictions of target binding sites of LOC100126784/miR-503-5p **(G)** and POM121L9P/miR-503-5p **(H)**. HEK293T cells co-transfected with miR-503-5p or control-mimics and luciferase reporter constructs containing WT or MUT of LOC100126784 **(I)** or POM121L9P **(J)**. ^∗^*p* < 0.05, ^∗∗∗^*p* < 0.001.

To clarify the intracellular localization of lncRNAs, respective RNA FISH probes for LOC100126784 and POM121L9P were designed. FISH (Figure 5A) and RT-qPCR analysis (Figure 5B) of nuclear and cytoplasmic RNAs revealed the abundant expression of cytoplasmic LOC100126784 and POM121L9P in BMSCs. To further study the regulatory relationships among LOC100126784, POM121L9P, and miR-503-5p, we performed an RIP assay for Ago2, the core component of the RNA-induced silencing complex. RT-qPCR (Figures 5C,E) and gel electrophoresis (Figures 5D,F) analyses showed that LOC100126784, POM121L9P and miR-503-5p were both markedly enriched by the anti-Ago2 antibody compared with the control anti-IgG antibody. Moreover, to confirm the direct binding sites between these lncRNAs and miR-503-5p, we generated dual-luciferase reporter WT-lncRNA and MUT-lncRNA plasmids. Co-transfection of the luciferase reporter plasmids containing WT-LOC100126784 plus miR-503-5p into BMSCs decreased reporter activity (Figures 5G,H). A similar difference in fluorescence activity was observed between WT-POM121L9P and MUT-POM121L9P plasmid transfection (Figures 5I,J). Thus, our results suggested that LOC100126784 and POM121L9P directly bind to miR-503-5p.

### Knockdown of *SORBS1* in Bone Marrow Mesenchymal Stem Cells Suppressed Osteogenic Differentiation

**FIGURE 6:**
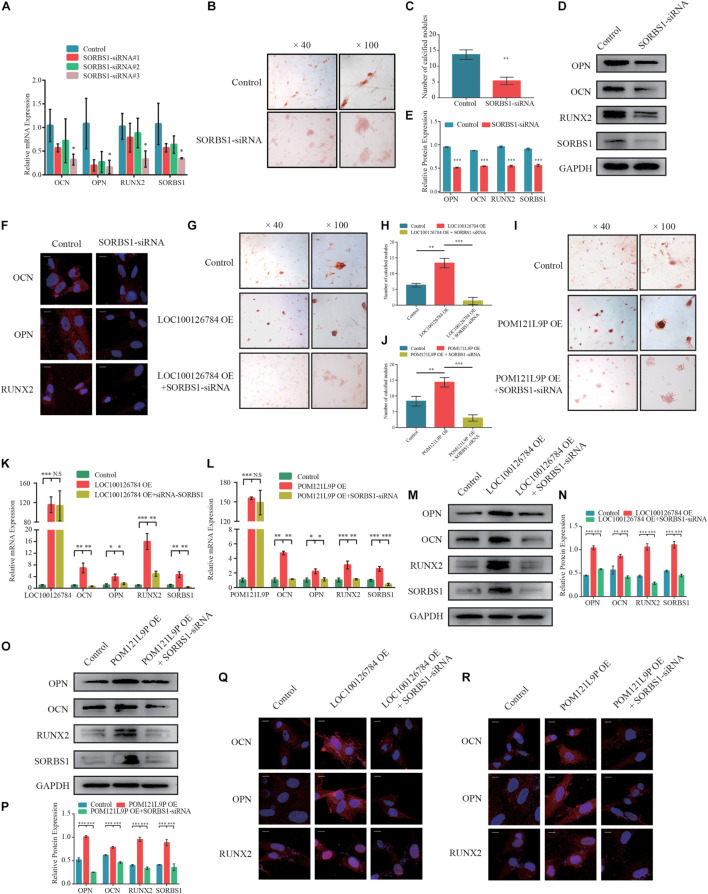
SORBS1 knockdown blocks early osteogenesis in BMSCs and is identified as the downstream of LOC100126784 and POM121L9P. **(A)** BMSCs were transfected with 100 nM SORBS1 or control siRNA. OCN, OPN, RUNX2, and SORBS1 expression levels as estimated by RT-qPCR. BMSCs were infected with control siRNA or SORBS1 siRNA#3, and then calcium nodes stained with Alizarin Red and observed at × 40 and × 100 magnification on day 7 of osteogenic differentiation **(B)**. **(C)** Quantitative analysis of the number of calcium nodules in **(B)**. Relative OCN, OPN, RUNX2, and SORBS1 protein expression levels as measured by western blotting **(D)**, quantified protein expression by densitometric analysis **(E)**, and immunofluorescence **(F)**. Then, BMSCs were transfected with LOC100126784- or POM121L9P- overexpressing lentiviral plasmids in the presence or absence of SORBS1 siRNA#3. Calcium nodes stained with Alizarin Red were observed and quantitative analysis was performed on day 7 during osteogensis **(G–J)**. The mRNA levels of OCN, OPN, RUNX2, and SORBS1 were detected by RT-qPCR **(K,L)**. Western blots with quantitative analysis **(M–P)** and IF assay **(Q,R)** were used to assess the protein expressions of OCN, OPN, RUNX2 and SORBS1. Quantitative data are presented as mean ± SD of three independent experiments. ^∗^*p* < 0.05, ^∗∗^*p* < 0.01, ^∗∗∗^*p* < 0.001.

To verify whether the effects of LOC100126784 and POM121L9P expression on osteogenesis stimulation were achieved through SORBS1, we first designed a loss-of-function experiment. Figure 6A shows that *SORBS1* knockdown with siRNA#3 decreased the mRNA levels of osteogenesis biomarkers. The number of calcified nodules stained Alizarin Red significantly decreased after *SORBS1* knockdown (Figures 6B,C). Similarly, *SORBS1* konckdown could downregulated the protein levels of OCN, OPN, and RUNX2, detected by Western blotting with quantitative analysis (Figures 6D,E) and IF (Figure 6F). In addition, after co-transfection with control plasmids, OE-LOC100126784, OE-POM121L9P, control-siRNA, and SORBS1-siRNA#3, Alizarin Red staining was used to assess mineralized nodule formation in BMSCs. LOC100126784 and POM121L9P overexpression promoted calcium nodule formation in BMSCs, whereas *SORBS1* silencing reversed osteogenic potential (Figures 6G–J). Moreover, the upregulation of mRNA (Figures 6K,L) and protein (Figures 6M–R) expression levels of the osteogenic biomarkers OCN, OPN, and RUNX2 after LOC100126784 or POM121L9P overexpression were counteracted in BMSCs with increasing SORBS1 expression.

**FIGURE 7:**
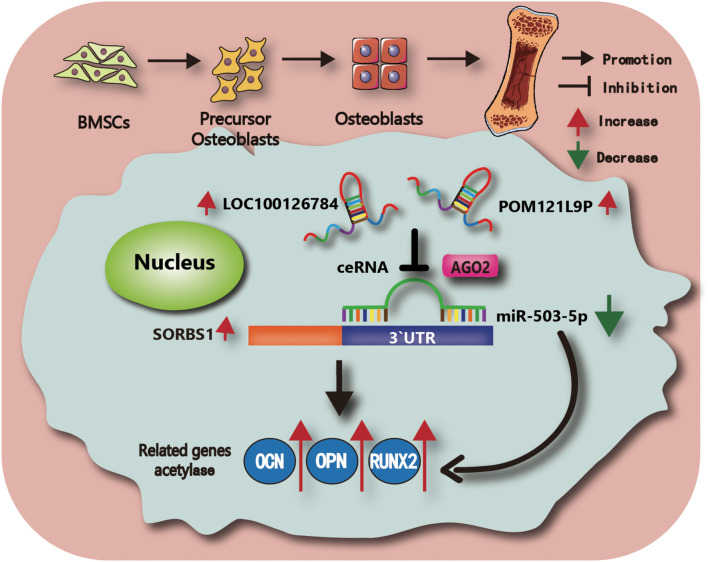
Schematic illustration of the working hypothesis. We hypothesize that LOC100126784 and POM121L9P derived from BMSCs can enhance osteogenic differentiation. These effects can be fulfilled through competitively sponging miR-503-5p with the AGO2-mediated silencing complex to release SORBS1.

Taken together, these findings revealed that miR-503-5p overexpression downregulated BMSC osteogenesis and *SORBS1*. Moreover, LOC100126784 and POM121L9P upregulation sponged miR-503-5p, alleviated *SORBS1* downregulation, and promoted osteogenic differentiation of BMSCs. The findings of the present study and the putative molecular mechanisms involved are summarized in Figure 7.

## Discussion

The bone marrow is a major site of osteogenic differentiation in mesenchymal stem cells (MSCs). Hence, it is an important research object in bone repair and osteoporosis therapy (Arthur et al., 2009). There is growing evidence that phenotype-specific regulation of osteogenesis occurs before day 7 of MSC induction (Kokabu et al., 2016; Robert et al., 2020). During this early period, osteogenic, adipogenic, or chondrogenic differentiation of BMSCs is determined by mechanisms regulating gene expression, transcription, and post-transcription. During this process, numerous non-coding (nc) RNAs extensively participate in post-transcriptional regulation (Schmitz et al., 2016; Hong et al., 2020). After osteogenic induction in human periodontal mesenchymal stem cells, lncRNA-POIR and miR-182 form a negative regulatory network comprising miR-182/FoxO1 (Wang et al., 2016). LncRNA-PCAT1 derived from human adipose-derived stem cells negatively regulates miR-145-5p and activates Toll-like receptor signaling (Yu et al., 2018). We hypothesized that a ceRNA network consisting of ncRNAs and mRNAs derived from BMSCs might induce osteogenic BMSC differentiation. The present study elucidated the differential expression profiles of lncRNAs, miRNAs, and mRNAs during BMSC osteogenesis. Through sequencing and bioinformatic analysis, we found that the expression levels of LOC100126784, POM121L9P, and SORBS1 were markedly increased in osteogenic-induced BMSCs on day 21, whereas the opposite expression patterns for miR-503-5p were observed. In addition, the presence of miR-503-5p binding sites in LOC100126784, POM121L9P, and SORBS1 sequences was validated by dual-luciferase and Ago2-RIP assays. Therefore, we propose that LOC100126784 and POM121L9P act as miR-503-5p sponges to relieve *SORBS1* downregulation and finally promote the osteogenic process in BMSCs.

miR-503-5p functions as a tumor suppressor in multiple cancers (Fu et al., 2019; Li et al., 2019; Wei et al., 2020). Moreover, as a mammal-specific member of the miR-15/107 miRNA family, miR-503-5p participates in stress response, tissue differentiation, and tissue remodeling (Jee et al., 2018; Wang et al., 2019; Shen et al., 2020). However, the direction of stem cell differentiation influenced by miR-503-5p remains unclear. Our findings showed that miR-503-5p decreased the expression of osteogenic differentiation markers OCN, OPN, and RUNX2. Further, according to the observed levels of osteogenic biomarkers and the number and size of calcium nodules on day 7, co-transfection with miR-503-5p abolished the intensified osteogenesis due to LOC100126784 or POM121L9P. Collectively, these results indicate that LOC100126784 and POM121L9P sponge miR-503-5p to modulate osteogenic differentiation at the early stages in induced BMSCs.

SORBS1, also known as CAP, participates in MSC differentiation depending on the stiffness of the ECM (Lin et al., 2001a, b; Kuroda et al., 2018). MSCs link ECM with focal adhesions and activate internal biochemical signaling pathways related to the cytoskeleton (Watt and Huck, 2013). They detect external mechanosensing and mechanotransduction. CAP promotes nuclear localization of YAP/TAZ on rigid (glass) ECM (Kuroda et al., 2017, 2018). Elevated YAP/TAZ ratios in the nucleus and cytoplasm favor the differentiation of osteoblasts, rather than adipoblasts, because of the direct interaction between TAZ and RUNX2 (Watt and Huck, 2013; Han et al., 2019). To clarify the role of SORBS1 in the regulation of osteogenic differentiation, we transfected BMSCs with SORBS1 siRNA and found relative decreases in osteogenic marker levels during early osteoblast differentiation. Further staining with Alizarin Red revealed that SORBS1 inhibition prevented early calcium nodule formation. However, the transfection of LOC100126784- and POM121L9P-overexpressing plasmids restored the mRNA and protein expression levels of the osteogenic markers OCN, OPN, and RUNX2, as well as SORBS1. Thus, we identified SORBS1 as the potential target of LOC100126784 and POM121L9P/miR-503-5p, providing a novel therapeutic target for osteogenic induction in BMSCs.

There are certain limitations to this study. The detailed mechanism of SORBS1 in BMSCs undergoing osteogenesis was not examined. The role of SORBS1 in determining whether MSCs undergo adipogenesis or osteogenesis remains to be elucidated. In addition, the mechanisms and effects of this process require *in vivo* verification. In future research, we plan to construct a rabbit tibia bone defect model to determine whether LOC100126784- and POM121L9P-OE adeno-associated viruses accelerate bone repair. Inhibition of this repair process by miR-503-5p will confirm the feasibility and potential clinical applicability of the ceRNA regulatory network.

In conclusion, this study showed that LOC100126784 and POM121L9P promote the progression of early osteogenic differentiation through the miR-503-5p-SORBS1 pathway. Our findings provide a theoretical basis for stem cell transplantation in the clinical treatment of osteoporosis and bone defects (Matsui and Corey, 2017).

## Data Availability Statement

The data presented in the study are deposited in the GEO repository, accession number GSE178679.

## Ethics Statement

The studies involving human participants were reviewed and approved by the Ethical Committee of The First Affiliated Hospital of Sun Yat-sen University (IRB: 2014C-028). The patients/participants provided their written informed consent to participate in this study. The animal study was reviewed and approved by The First Affiliated Hospital of Sun Yat-sen University ([2013]A-110) Animal Research Committee.

## Author Contributions

GM and YK contributed to the study conception and design. YX, RX, HS, DL, ZL, HL, ZZ, and TX performed the experiments. YX and RX analyzed the data, prepared the figures, and wrote the manuscript. GM and ZZ revised the manuscript. YK participated in the discussion of the manuscript. All authors contributed to the article and approved the submitted version.

## Conflict of Interest

The authors declare that the research was conducted in the absence of any commercial or financial relationships that could be construed as a potential conflict of interest.

## Publisher’s Note

All claims expressed in this article are solely those of the authors and do not necessarily represent those of their affiliated organizations, or those of the publisher, the editors and the reviewers. Any product that may be evaluated in this article, or claim that may be made by its manufacturer, is not guaranteed or endorsed by the publisher.

## Footnotes

1
www.bio-rad.com

## Supplementary Material

Supplementary Figure 1 Knockdown of LOC100126784 and POM121L9P inhibits osteogenesis but the effect is reversed by miR-503-5p-inhibitor. **(A)** BMSCs were transfected with miR-503-5p inhibitor or miR-inhibitor-control at 50 nM. The expression level of miR-503-5p as measured by RT-qPCR, 48 h after transfection. **(B)** siRNA- LOC100126784, siRNA- POM121L9P, and control siRNA were transfected with 100 nM. The knockdown efficiency LOC100126784 and POM121L9P as examined by RT-qPCR. BMSCs were infected with siRNA- LOC100126784#1, siRNA- POM121L9P#1, and control siRNA in the presence or absence of miR-503-5p inhibitor or miR-inhibitor-control. Relative OCN, OPN, RUNX2, and SORBS1 expression levels as estimated using RT-qPCR **(D–F)**, western blots **(G–I),** and IF **(J–L)**. Quantitative data are presented as mean ± SD of three independent experiments. ^∗^*p* < 0.05, ^∗∗^*p* < 0.01, ^∗∗∗^*p* < 0.001.
