# Supplementary figures and images for: Long Non-coding RNAs LOC100126784 and POM121L9P Derived From Bone Marrow Mesenchymal Stem Cells Enhance Osteogenic Differentiation via the miR-503-5p/SORBS1 Axis
Source: Front Cell Dev Biol. 2021 Oct 22;9:723759. doi: 10.3389/fcell.2021.723759 (PMC8570085; doi:10.3389/fcell.2021.723759)

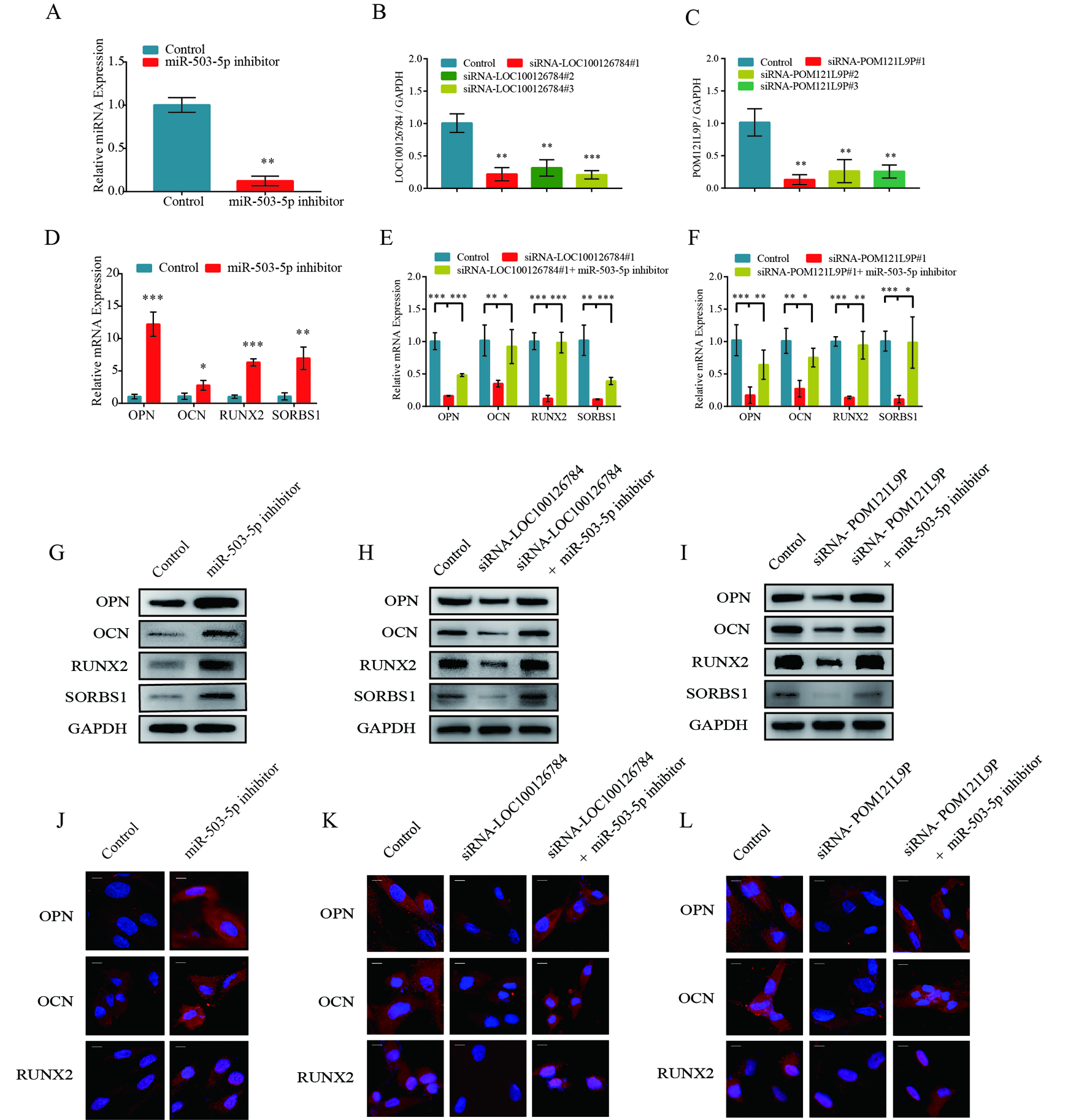

Supplement: Supplementary Figure 1 — Knockdown of LOC100126784 and POM121L9P inhibits osteogenesis but the effect is reversed by miR-503-5p-inhibitor. (A) BMSCs were transfected with miR-503-5p inhibitor or miR-inhibitor-control at 50 nM. The expression level of miR-503-5p as measured by RT-qPCR, 48 h after transfection. (B) siRNA- LOC100126784, siRNA- POM121L9P, and control siRNA were transfected with 100 nM. The knockdown efficiency LOC100126784 and POM121L9P as examined by RT-qPCR. BMSCs were infected with siRNA- LOC100126784#1, siRNA- POM121L9P#1, and control siRNA in the presence or absence of miR-503-5p inhibitor or miR-inhibitor-control. Relative OCN, OPN, RUNX2, and SORBS1 expression levels as estimated using RT-qPCR (D–F), western blots (G–I), and IF (J–L). Quantitative data are presented as mean ± SD of three independent experiments. ∗p < 0.05, ∗∗p < 0.01, ∗∗∗p < 0.001. [file Image_1.TIF]

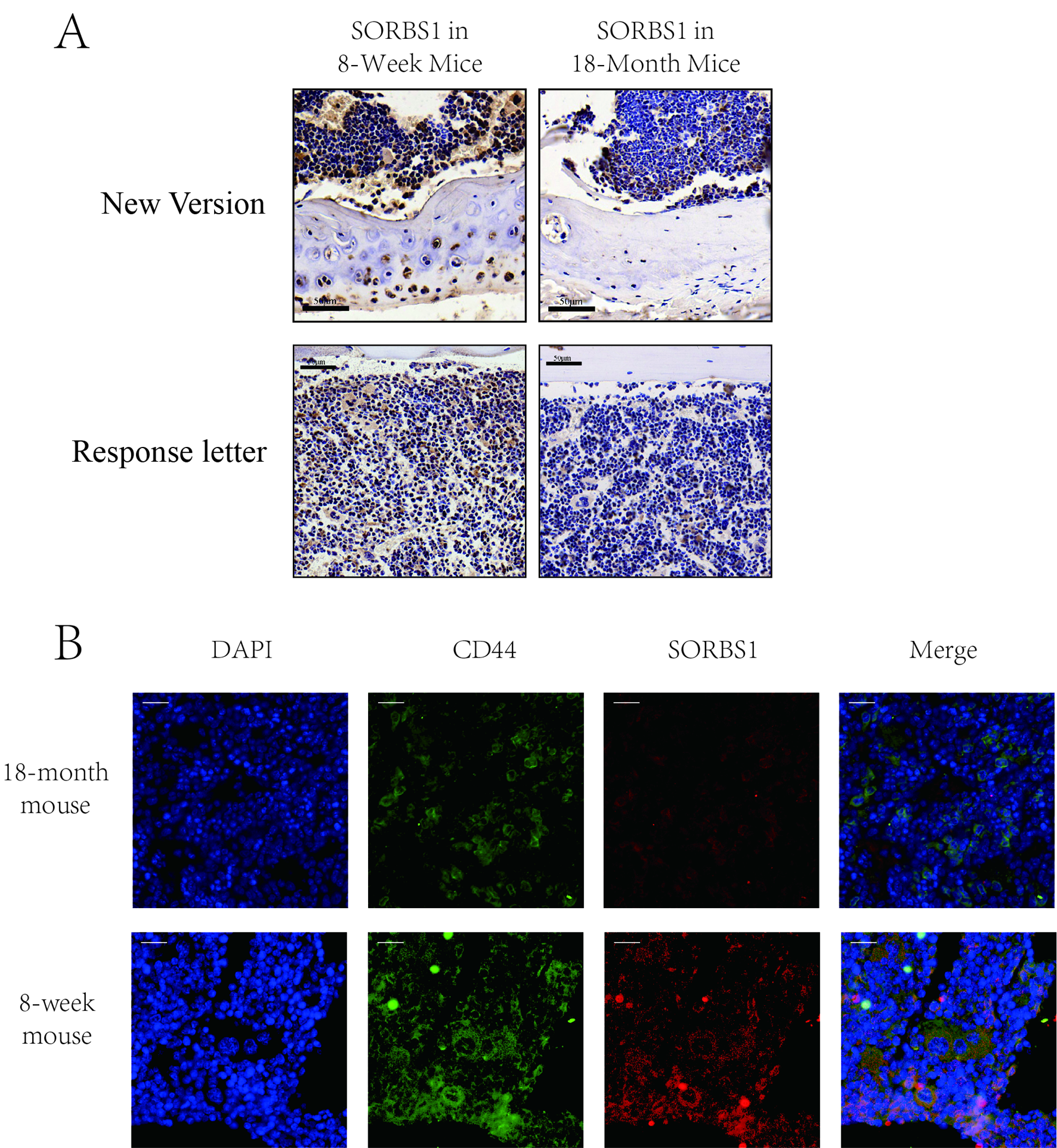

Supplement: Supplementary file 2 [file Image_2.TIF]
