# Supplementary material for: Long Non-coding RNAs LOC100126784 and POM121L9P Derived From Bone Marrow Mesenchymal Stem Cells Enhance Osteogenic Differentiation via the miR-503-5p/SORBS1 Axis
Source: Front Cell Dev Biol. 2021 Oct 22;9:723759. doi: 10.3389/fcell.2021.723759 (PMC8570085; doi:10.3389/fcell.2021.723759)
Supplement: Supplementary file 3 [file Table_1.DOCX]

Supplementary Table 1

| Primers | Sequence（5‘ to 3’） |
| --- | --- |
| hsa-GAPDH-F | GCACCGTCAAGGCTGAGAAC |
| hsa-GAPDH-R | TGGTGAAGACGCCAGTGGA |
| hsa-OCN-F | AGGGCAGCGAGGTAGTGA |
| hsa-OCN-R | CCTGAAAGCCGATGTGGT |
| hsa-OPN-F | ATCTCCTAGCCCCACAGAAT |
| hsa-OPN-R | CATCAGACTGGTGAGAATCATC |
| hsa-Runx2-F | GGAGCGGACGAGGCAAGAGT |
| hsa-Runx2-R | AGGAATGCGCCCTAAATCAC |
| hsa-SORBS1-F | ACAGAGGGCAAGAAGGC |
| hsa-SORBS1-R | TCGGGAAGGTCAGAGGT |
| hsa-POM121L9P-F | TGGCAGAGGGGTCACAG |
| hsa-POM121L9P-R | AGCTTCGCAGAGGGCAT |
| hsa-LOC100126784-F | AGCAACCCAAACAGCGA |
| hsa-LOC100126784-R | CTGAAGAAGCCGGGAGAG |
| hsa-miR-503-5p | TAGCAGCGGGAACAGTTCTGCAG |
| hsa-miR-4443 | TTGGAGGCGTGGGTTTT |
| hsa-miR-210-5p | AGCCCCTGCCCACCGCACACTG |
| hsa-miR-615-3p | TCCGAGCCTGGGTCTCCCTCTT |
| hsa-miR-3928-3p | GGAGGAACCTTGGAGCTTCGGC |
| hsa-U6-F | CTCGCTTCGGCAGCACA |
| hsa-U6-R | AACGCTTCACGAATTTGCGT |
